# Social Risk Factors for an Injury in Paralympic Athletes: Examining Time to Access the Training Facility and Time to Prepare Before and After Training

**DOI:** 10.1177/23259671251320986

**Published:** 2025-06-06

**Authors:** Maxime Luiggi, Rémi Richard, Valentine Duquesne, Hélène Joncheray

**Affiliations:** † Apprentissage, Didactique, Evaluation, Formation Laboratory, Aix-Marseille University, Marseille, France; ‡ Sciences Economiques & Sociales de la Santé & Traitement de l’Information Médicale, Institute of Public Health Sciences, Aix-Marseille University, Marseille, France; § Santesih Research Unit, University of Montpellier, Montpellier, France; ‖ French Paralympic and Sports Committee, Paris, France; ¶ Sport, Expertise and Performance Laboratory, National Institute of Sport, Expertise, and Performance, Paris, France; # Institut des Sciences du Sport Santé de Paris, Paris Cité University, Paris, France; Investigation performed at the Sport, Expertise and Performance Laboratory, National Institute of Sport, Expertise, and Performance, Paris, France and the Santesih Reseach Unit, University of Montpellier, Montpellier, France

**Keywords:** epidemiology, injury prevention, sports injury, disability, Paralympic, social factors

## Abstract

**Background:**

No previous study has analyzed the associations between Paralympic athletes’ sociocultural factors and injury risk.

**Purpose/Hypothesis:**

The purpose of this study was to examine the associations of time to access the main training facility (TAF) and time to prepare before and after training (TPT) with the injury risk while controlling for sport, impairment type, and performance level. It was hypothesized that a longer TAF and TPT would be associated with an increased injury risk.

**Study Design:**

Cross-sectional study; Level of evidence, 3.

**Methods:**

A retrospective questionnaire was sent to 186 French athletes in 11 Paralympic sports who were identified by their sports federations as qualified to compete at the 2021 Tokyo Paralympic Games. The level of performance was determined according to past performance in international championships and the Paralympic Games. Impairment type was self-reported by athletes and then reclassified by a certified physician (limb deficiency, visual impairment, spinal cord–related disorder, central neurological disorder, short stature, peripheral neurological and muscular disorder). TAF and TPT were assessed by 2 self-reported questions. Injuries in the past 12 months were assessed using the latest consensus statement for the recording and reporting of self-reported epidemiological data on injuries in sports. Logistic regression analyses were performed to evaluate the relationship of TAF and TPT with the injury risk while controlling for sport, impairment type, and performance level.

**Results:**

A total of 126 athletes (response rate: 67.7%) agreed to participate. The prevalence of injuries was 35.7% (95% CI, 33.3%-38.2%); it was lower among athletes with a high level of performance compared with those with a low level of performance. Athletes who reported a TAF ≥40 minutes were 5.5 times (95% CI, 1.2-30.5) more likely to have sustained an injury than those who reported <10 minutes. Athletes who reported a TPT ≥1 hour and 30 minutes were 6.3 times (95% CI, 1.1-44.4) more likely to have had an injury than those who reported 15 minutes.

**Conclusion:**

Our study demonstrated that Paralympic athletes who reported a longer TAF and TPT had a higher risk of injuries. Future studies are needed to understand the exact mechanism that would explain these results and to assist in the prevention of injuries in this population of athletes.

Paralympic athletes have a greater risk of injuries compared with Olympic athletes.^4^ Identifying the extent and severity as well as the risk factors and mechanisms of an injury represents the first steps for the prevention of sports injuries. Thereafter, prevention programs can be developed.^26^

According to Bahr and Krosshaug’s^2^ comprehensive model for injury causation in sports, risk factors for an injury can be defined as internal or external. Internal risk factors include athlete characteristics predisposing them to the occurrence of an injury (eg, history of injuries or physical fitness).^13,27^ External risk factors include environmental characteristics of sports participation that increase the injury risk (eg, type of sport or playing position).^3,17^ In the biopsychosocial sports injury risk profile proposed by Wiese-Bjornstal,^28^ internal and external risk factors are divided into 2 different categories: biological/psychological and physical/sociocultural, respectively.

Within the few epidemiological studies of sports injuries conducted among Paralympic athletes, only 1 internal biological risk factor (impairment type) and 1 external physical risk factor (type of sport) have been routinely identified.^5,7,25^ These studies have consistently shown that athletes with a limb deficiency had the highest risk of injuries, while those with a neurological impairment had the lowest.^5,7,25^ As for sport type, five-a-side football, as well as team and contact sports, has repeatedly been identified as having a high risk of injuries.^5,19^ Only 1e study has found other risk factors including biological (age, use of medication), physical (use of assistive device), and psychological (anxiety/depression symptoms) factors using univariate analyses.^7^

According to the Human Development Model–Disability Creation Process (HDM-DCP), disability is the result of an interaction between personal factors (such as age, sex, medical condition) and environmental factors (social and/or physical).^9,10^ An incompatibility between personal and environmental factors can result in challenges in daily life such as difficulties with transportation or preparation. We argue that these challenges can contribute to increased overall daily stress levels, which could further heighten the risk of injuries.^15,21,29^ More specifically, longer transportation times can contribute to overall daily stress because of travel constraints, which is particularly relevant for disabled athletes who often encounter environments poorly adapted to their needs (eg, visual aids for visually impaired or ramps and lifts for wheelchair users or those with impaired mobility).^20^ For instance, a study on a nonsporting population of wheelchair users found that prolonged wheelchair use was associated with an increased risk of upper extremity pain and injuries.^18^ It is plausible that similar factors, such as extended transportation times involving multiple transitions between different modes of transport (eg, switching from subway to bus), could also elevate the injury risk among Paralympic athletes. Similar to transportation time, a longer preparation time might serve as a proxy for the overall difficulties and constraints that athletes face in performing fundamental daily tasks. These difficulties may impair their training ability through various mechanisms (eg, reduced attention, increased fatigue), potentially increasing the injury risk.^14,29^

In this study, we aimed to investigate the social factors of Paralympic athletes’ everyday lives that may impact their injury risk, which have not been investigated yet. The main objective of this study was to examine the associations of time to access the main training facility (TAF) and time to prepare before and after training (TPT) with the injury risk in Paralympic athletes while controlling for other risk factors for an injury identified in the sports medicine literature (sport, type of impairment, level of performance). It was hypothesized that a longer TAF and TPT would be associated with an increased injury risk.

## Methods

This study is part of a larger project funded by the French National Research Agency (ANR) and entitled “PARAPERF: Optimizing Paralympic Performance: From Identification to Medal Winning”, aimed at identifying performance factors used by athletes in 11 Paralympic sports. Part of this project included an online questionnaire asking athletes about their sociodemographic characteristics, sport, level of performance, training organization (including sociocultural factors), and injuries. This study has been approved by the ethical review board of the funding organization (grant No. ANR-19-STPH-005). This study was also approved by the national directors of the sports federations involved in this project (n = 2 [responsible for 11 Paralympic sports]).

Before participation, the athletes were informed of the purpose of the study as well as the potential benefits and risks associated with participation. A disclosure statement guaranteed the anonymity of answers and their use only for research purposes. The protocol for this research was drawn up in compliance with the General Data Protection Regulation. An informed consent form was included at the beginning of the questionnaire, specifying the modalities of data anonymization and use.

### Participants and Questionnaire

The online questionnaire was developed using LimeSurvey between July 15 and October 22, 2020, in collaboration between researchers and staff members of the sports federations. French athletes in archery, athletics, boccia, cycling, fencing, five-a-side football, powerlifting, rugby, shooting, swimming, and table tennis who were identified by their sports federations as qualified to compete at the 2021 Tokyo Paralympic Games (n = 186) were invited to fill out the questionnaire anonymously between October 23, 2020, and February 3, 2021. Multiple reminders to participate were made by the sports federations and researchers over this period (n = 8). To fill out the questionnaire, the athletes were able to ask for help from a third party if their health condition required it.

Data were collected at 5 months after the confinement period, from March 17 to May 11, because of the COVID-19 pandemic. To minimize potential response biases related to this period, athletes were instructed at the beginning of the survey to base their answers on their usual training and living habits, excluding the exceptional conditions that they experienced during the spring 2020 confinement period.

### Variables of Interest

#### Sport

Athletes were invited to specify their sport (archery, athletics, boccia, cycling, fencing, five-a-side football, powerlifting, rugby, shooting, swimming, table tennis). The final sample was compared to the French population of Paralympic athletes. Response rates per sport were 72.7% for archery, 54.9% for athletics, 75.0% for boccia, 65.5% for cycling, 80.0% for fencing, 50.0% for five-a-side football, 50.0% for powerlifting, 81.8% for rugby, 90.0% for shooting, 66.7% for swimming, and 72.2% for table tennis.

#### Impairment Type

Questions were developed to categorize athletes according to the impairment classification systems used in previous studies of Paralympic athletes.^5^ Athletes were invited to report their impairment as a predefined type: intellectual and/or psychic, neurological (central, peripheral, neuromuscular, or other), orthopaedic, visual, or other. Those answering “other” were asked to further describe their impairment in a text box.

Second, the cause of their impairment was detailed: stroke, spinal cord injury leading to paraplegia, spinal cord injury leading to tetraplegia, multiple sclerosis, head injury, cerebral palsy, Charcot-Marie-Tooth disease, myopathy or spinal amyotrophy, brachial plexus palsy, left peroneal nerve palsy, lower limb hemimelia, upper limb hemimelia, amputation, limb length inequality (upper, lower, or both), short stature, osteogenesis imperfecta, autism spectrum disorder, or other. Those answering “other” were asked to further describe the cause of their impairment in a text box. Finally, all participants were free to describe their impairment in their own words.

With the data gathered, we and a certified physician in physical medicine and rehabilitation reclassified each participant appropriately into 1 of the following impairment types as previously mentioned by Derman et al^5^: limb deficiency (n = 32), visual impairment (n = 16), spinal cord–related disorder (n = 43), central neurological disorder (n = 16), short stature (n = 1), peripheral neurological and muscular disorder (n = 13), and unknown/other (n = 5). Only 1 athlete declared a short stature and was therefore placed in the unknown/other category. All impairment types according to the different categorization systems used are available in Appendix Table A1.

#### Level of Performance

The level of performance was determined using the Australian Institute of Sport’s model of athletes’ development (https://www.ais.gov.au/ftem).^12^ The Foundations, Talent, Elite and Mastery framework involves 4 macrophases of an athlete’s development, which are subdivided into 10 microphases.

The Foundations stage is the stage in which athletes acquire the fundamentals of the sport. The Talent stage is the stage in which athletes are identified by the sports institutions in charge of performance as those likely to achieve high levels of performance in the short or long term. The Elite stage corresponds to the stage in which athletes represent their country in international competitions and potentially achieve podium finishes. Within the Elite stage, a distinction is made between athletes who have participated in international competitions without winning medals (E1) and those who have attained 1 to 3 medals (E2). The Mastery stage represents the stage in which athletes have won multiple international medals in at least the last 4 years. This last stage corresponds to athletes who dominate their discipline by being among the top 3 in the world for a significant number of years.

The minimum level of development of the athletes was the Talent stage, as they were all identified by their sports federations to compete at the 2021 Tokyo Paralympic Games and benefited from specific support for training and competitions. To classify the athletes in the 3 upper stages, they were invited to report whether they had participated in the (1) 2017, 2018, and 2019 World Championships; (2) 2017, 2018, and 2019 European Championships; and (3) Paralympic Games since Seoul (1988). If they answered “yes,” they were invited to report their results (did not participate in the final phase, participated in the final phase, third position, second position, first position).

The sample included 22 Talent athletes (17.5%), 31 E1 athletes (24.6%), 59 E2 athletes (46.8%), and 14 Mastery athletes (11.1%). To obtain a more balanced distribution, the E2 stage was divided into 2 different levels. E2– athletes were those who won 1 medal in an international competition, while E2+ athletes were those who won 2 or 3 medals in international competitions. The final sample included 22 Talent (17.5%), 31 E1 (24.6%), 33 E2– (26.2%), 26 E2+ (20.6%), and 14 Mastery (11.1%) athletes. The final sample was compared to the French population of Paralympic athletes. Response rates per category were 75.9% for Talent, 58.5% for E1, 70.2% for E2–, 70.3% for E2+, and 70.0% for Mastery.

Table 1 shows the sample distribution according to level of performance and sport. It demonstrates that Talent athletes were represented in 4 of 11 sports: five-a-side football, cycling, rugby, and athletics. Mastery athletes were represented in 4 sports: table tennis, fencing, swimming, and athletics. In table tennis, 61.5% of the athletes were Mastery. By contrast, in five-a-side football, 50.0% of the athletes were Talent.

**Table 1:** Sport by Level of Performance^*a*^

	T (n = 22)	E1 (n = 31)	E2– (n = 33)	E2+ (n = 26)	M (n = 14)	All (n = 126)
Athletics	6 (21.4)	4 (14.3)	7 (25.0)	8 (28.6)	3 (10.7)	28 (100.0)
Boccia	0 (0.0)	0 (0.0)	3 (100.0)	0 (0.0)	0 (0.0)	3 (100.0)
Cycling	9 (47.4)	6 (31.6)	4 (21.1)	0 (0.0)	0 (0.0)	19 (100.0)
Fencing	0 (0.0)	4 (33.3)	1 (8.3)	5 (41.7)	2 (16.7)	12 (100.0)
Five-a-side football	3 (50.0)	1 (16.7)	0 (0.0)	2 (33.3)	0 (0.0)	6 (100.0)
Powerlifting	0 (0.0)	0 (0.0)	2 (100.0)	0 (0.0)	0 (0.0)	2 (100.0)
Swimming	0 (0.0)	2 (25.0)	2 (25.0)	3 (37.5)	1 (12.5)	8 (100.0)
Rugby	4 (22.2)	3 (16.7)	6 (33.3)	5 (27.8)	0 (0.0)	18 (100.0)
Table tennis	0 (0.0)	1 (7.7)	2 (15.4)	2 (15.4)	8 (61.5)	13 (100.0)
Shooting	0 (0.0)	4 (44.4)	4 (44.4)	1 (11.1)	0 (0.0)	9 (100.0)
Archery	0 (0.0)	6 (75.0)	2 (25.0)	0 (0.0)	0 (0.0)	8 (100.0)

a Data are presented as n (%). E1, Elite 1; E2–, Elite 2–; E2+, Elite 2+; M, Mastery; T, Talent.

#### Sports Injury and Injury Severity

We defined a sports injury as “any injury or musculoskeletal pain that caused changes in normal training or competition to the mode, duration, intensity, or frequency, regardless of whether or not time is lost from training or competition.”^8^ The athletes were invited to report whether they had a sports injury during the 2019-2020 season. Those who answered “yes” were asked to indicate how many sports injuries (ASIs) that they had during this time period. The 12-month recall period was chosen because previous studies have shown 100% accuracy in athletes reporting at least 1 injury versus no injury.^11^

Injury severity was determined through (1) time loss from training and competitions and (2) the degree of medical attention received by the athlete (no medical attention, treated by a medical practitioner, hospitalization). The degree of medical attention was divided into 2 levels: ASI including all injury severities and medically treated injury (MTI) including injuries treated by a medical practitioner or that led to hospitalization.

Injury severity was only asked for 1 injury. If the athletes had multiple injuries in the past season, they were asked to report the severity only for the injury that caused the biggest change in their usual training in terms of volume, intensity, and time loss from their sports participation. Athletes were invited to report the location and nature of this injury. However, this information was not included in the present study, as previous studies have shown that these details were not sufficiently accurate using self-report methodologies.^1^

#### Sociocultural Factors

##### Access Time (TAF)

Participants were invited to answer the following question by ticking the appropriate box (<10 minutes, 10-19 minutes, 20-39 minutes, ≥40 minutes): “On average, how long does it take altogether to go to your main training facility?” Time categories were selected based on the expertise of one author (V.D.), who is responsible for the socioprofessional supervision of Paralympic athletes and has extensive knowledge of Paralympic training conditions.

##### Preparation Time (TPT)

Participants were invited to answer the following question by ticking the appropriate box (15 minutes, 30 minutes, 1 hour, ≥1 hour 30 minutes): “On average, how long does it take to get prepared before and after your training (get dressed, change wheelchair, take a shower, etc)?” Time categories were selected based on the expertise of 1e author, who is responsible for the socioprofessional supervision of Paralympic athletes and has extensive knowledge of Paralympic training conditions.

### Statistical Analysis

Analyses were conducted using the open-source statistical software R (Version 3.6.4).

#### Descriptive Analysis

First, the ASI and MTI prevalence by level of performance, sport, impairment type, TAF, and TPT were computed. For descriptive statistics, we determined 95% confidence intervals (CIs) for the cohort of Paralympic athletes surveyed. In this context, the 95% CIs were computed based on the survey response rate. Specifically, if the response rate was 100%, the 95% CIs would be equal to the computed proportions. Consequently, these 95% CIs are limited to the examined population in France and cannot be generalized to a larger population, such as Paralympic athletes from multiple countries. This approach is suitable for the present study, given that training and life habits are assumed to vary across countries, thereby restricting the results to the specific population of Paralympic athletes examined. We used the following formula:

$$e=Z_{\alpha }\sqrt{\frac{p(1-p)}{n}}\sqrt{\frac{N-n}{N-1}}$$

where *Z* = 1.96 is the *Z* score for the 95% CI, *p* is the observed proportion of the specified outcome, *n* is the sample size (ie, 126), and *N* is the population size (ie, 186).

When the exact response rates for specific categories were available (eg, for level of performance or sport), we computed the 95% CI using the corresponding response rate for those categories. In cases in which exact response rates were not available (eg, for impairment type, TAF, and TPT), we used the overall survey response rate to compute the 95% CI. For statistical comparisons between variable categories, we used the 95% CI, considering differences statistically significant at the 0.05 level when the 95% CIs for the categories did not overlap.

Second, the bivariate associations between level of performance, sport, and impairment type were presented. Third, the distributions of TAF and TPT were presented by level of performance, sport, and impairment type.

#### Multivariable Analysis

First, multiple univariate binary logistic regression models were performed to examine the relationship between all variables and ASIs. Second, a multivariable binary logistic regression model was performed to assess the relationship between TAF and TPT while controlling for level of performance, sport, and impairment type. Third, an additional model using the forward and backward stepwise technique was performed. The goal was to create the simplest regression model that best fit the study data. Model fit was compared using the Akaike information criterion and the area under the curve. The model was chosen according to these latter parameters, assuming that (1) the lower the Akaike information criterion, the better the model, (2) and the higher the area under the curve, the better the model.

To respect the rule of 10 events per variable in the use of logistic regression models, the 11 sports were recategorized into 6 categories.^22^ The categories were formed to reach the minimum number of 10 athletes per category while grouping the most similar sports in terms of physical activity intensity and level of contact. These 2 parameters are those used by the Council on Sports Medicine and Fitness to examine the injury risk between sports.^23^ The final 6 categories were as follows: athletics/powerlifting (high intensity/low contact; n = 30), archery/boccia/shooting (low intensity/low contact; n = 20), cycling/swimming (high intensity/low contact; n = 27), fencing (n = 12), five-a-side football/rugby (high intensity/high contact; n = 24), and table tennis (n = 13).

Concerning the impairment type, only 6 athletes were in the unknown/other category. No further information concerning their impairment was available to group them with other impairment types. A statistical procedure was thus chosen for that purpose. After descriptive analyses, they were reclassified in the impairment type that was the closest in terms of injury prevalence. Finally, a supplemental statistical model was performed by adding paraplegia/tetraplegia to evaluate whether the results obtained differed significantly from analyses in the second and third steps.

## Results

A total of 126 athletes completed the survey entirely (response rate: 67.7%). No missing responses were present because they were not allowed while filling out the questionnaire. The mean time to answer the survey was 27 minutes. Overall, 35.7% (n = 45) (95% CI, 33.3%-38.2%) of Paralympic athletes reported an ASI. Among these 45 Paralympic athletes, 32 reported a time loss <21 days, 5 reported between 21 days and 3 months, and 8 reported >3 months. In addition, 11 did not have their injury medically treated, 31 had an MTI without hospitalization, and 3 were hospitalized after their injury. Table 2 shows the prevalence of ASIs and MTIs by level of performance, sport, impairment type, TAF, and TPT.

**Table 2:** Level of Performance, Sport, Impairment Type, TAF, and TPT by Injury Severity^*a*^

	Response Rate, %	ASI		MTI	
		n (%)	95% CI^*b*^	n (%)	95% CI^*b*^
Level of performance^*c*^					
T (n = 22)	75.9	10 (45.5)	35.1-55.9	4 (18.2)	10.1-26.2
E1 (n = 31)	58.5	10 (32.3)	21.6-43.0	2 (6.5)	0.8-12.1
E2– (n = 33)	70.2	13 (39.4)	30.2-48.6	5 (15.2)	8.4-21.9
E2+ (n = 26)	70.3	10 (38.5)	28.1-48.8	2 (7.7)	2.0-13.4
M (n = 14)	70.0	2 (14.3)	4.0-24.6	0 (0.0)	0.0-21.5
Sport^*c*^					
Athletics (n = 28)	54.9	16 (57.1)	44.7-69.6	4 (14.3)	5.5-23.1
Boccia (n = 3)	75.0	0 (0.0)	0.0-56.2	0 (0.0)	0.0-56.2
Cycling (n = 19)	65.5	3 (15.8)	6.0-25.6	1 (5.3)	0.0-11.3
Fencing (n = 12)	80.0	6 (50.0)	36.9-63.1	3 (25.0)	13.7-36.3
Five-a-side football (n = 6)	50.0	1 (16.7)	0.0-38.7	1 (16.7)	0.0-38.7
Powerlifting (n = 2)	50.0	1 (50.0)	0.0-100.0	1 (50.0)	0.0-100.0
Swimming (n = 8)	66.7	0 (0.0)	0.0-32.4	0 (0.0)	0.0-32.4
Rugby (n = 18)	81.8	8 (44.4)	34.4-54.5	2 (11.1)	4.8-17.5
Table tennis (n = 13)	72.2	3 (23.1)	10.7-35.5	0 (0.0)	0.0-22.8
Shooting (n = 9)	90.0	4 (44.4)	33.6-55.3	1 (11.1)	4.3-18.0
Archery (n = 8)	72.7	3 (37.5)	19.1-55.9	0 (0.0)	0.0-32.4
Impairment type^*d*^					
Limb deficiency (n = 32)	67.7	11 (34.4)	24.9-43.8	4 (12.5)	5.9-19.1
Visual impairment (n = 16)	67.7	7 (43.8)	29.6-57.9	4 (25.0)	12.7-37.3
Spinal cord–related disorder (n = 43)	67.7	21 (48.8)	40.3-57.4	4 (9.3)	4.3-14.3
Central neurological disorder (n = 16)	67.7	6 (37.5)	23.7-51.3	1 (6.3)	0.0-13.1
Peripheral neurological and muscular disorder (n = 13)	67.7	0 (0.0)	0.0-22.8	0 (0.0)	0.0-22.8
Unknown/other (n = 6)	67.7	0 (0.0)	0.0-39.0	0 (0.0)	0.0-39.0
TAF^*d*^					
<10 min (n = 31)	67.7	4 (12.9)	6.1-19.7	1 (3.2)	0.0-6.8
10-19 min (n = 23)	67.7	8 (34.8)	23.6-46.0	2 (8.7)	2.1-15.3
20-39 min (n = 34)	67.7	13 (38.2)	28.9-47.6	4 (11.8)	5.6-18.0
≥40 min (n = 38)	67.7	20 (52.6)	43.5-61.7	6 (15.8)	9.1-22.4
TPT^*d*^					
15 min (n = 30)	67.7	4 (13.3)	6.4-20.3	1 (3.3)	0.0-7.0
30 min (n = 43)	67.7	19 (44.2)	35.7-52.7	6 (14.0)	8.0-19.9
1 h (n = 34)	67.7	13 (38.2)	28.9-47.6	4 (11.8)	5.6-18.0
≥1 h 30 min (n = 19)	67.7	9 (47.4)	34.4-60.4	2 (10.5)	2.6-18.5
All^*c*^ (n = 126)	67.7	45 (35.7)	33.3-38.2	13 (10.3)	8.8-11.9

a ASI, any sports injury; E1, Elite 1; E2–, Elite 2–; E2+, Elite 2+; M, Mastery; MTI, medically treated injury; T, Talent; TAF, time to access main training facility; TPT, time to prepare before and after training.

b The 95% CIs were computed considering the response rates per variable category.

c Response rates were determined.

d Response rates were assumed according to the overall survey response rate.

Concerning the level of performance, the ASI and MTI prevalence were the lowest among Mastery athletes (14.3% and 0.0%, respectively). However, they were the highest among Talent athletes (45.5% and 18.2%, respectively). Comparisons of the 95% CI showed that the prevalence of ASIs was statistically significantly lower among Mastery athletes (95% CI, 4.0%-24.6%) compared with E2+ (95% CI, 28.1%-48.8%), E2– (95% CI, 30.2%-48.6%), and Talent (95% CI, 35.1%-55.9%) athletes.

Concerning the sport, comparisons of the 95% CI showed that the prevalence of ASIs was statistically significantly lower in cycling (95% CI, 6.0%-25.6%) compared to athletics (95% CI, 44.7%-69.6%), fencing (95% CI, 36.9%-63.1%), rugby (95% CI, 34.4%-54.5%), and shooting (95% CI, 33.6%-55.3%). The ASI prevalence in athletics (95% CI, 44.7%-69.6%) was statistically significantly higher compared to cycling (95% CI, 6.0%-25.6%), fencing (95% CI, 36.9%-63.1%), five-a-side football (95% CI, 0.0%-38.7%), swimming (95% CI, 0.0%-32.4%), and table tennis (95% CI, 10.7%-35.5%). Comparisons of the 95% CI showed that the prevalence of ASIs was statistically significantly lower in cycling (95% CI, 6.0%-25.6%) compared to athletics (95% CI, 44.7%-69.6%), fencing (95% CI, 36.9%-63.1%), rugby (95% CI, 34.4%-54.5%), and shooting (95% CI, 33.6%-55.3%). The ASI prevalence in athletics (95% CI, 44.7%-69.6%) was statistically significantly higher compared to cycling (95% CI, 6.0%-25.6%), fencing (95% CI, 36.9%-63.1%), five-a-side football (95% CI, 0.0%-38.7%), swimming (95% CI, 0.0%-32.4%), and table tennis (95% CI, 10.7%-35.5%).

Concerning the impairment type, no athlete with a peripheral neurological and muscular disorder had sustained an injury in the past 12 months. The ASI prevalence increased gradually in athletes with a limb deficiency (34.4%), athletes with a central neurological disorder (37.5%), athletes with a visual impairment (43.8%), and athletes with a spinal cord–related disorder (48.8%). Comparisons of the 95% CI showed that the prevalence of ASIs was statistically significantly lower for athletes with a peripheral neurological and muscular disorder compared to athletes with other impairment categories.

It should be noted that the MTI prevalence was only partially correlated with the ASI prevalence. Hence, athletes with a spinal cord–related disorder had the highest prevalence of ASIs (48.8%), but only 9.3% had sustained an MTI. Contrarily, athletes with a limb deficiency were the second lowest subgroup in terms of the ASI prevalence (34.4%), but 12.5% of them had sustained an MTI. This prevalence reached 25.0% among athletes with a visual impairment.

Concerning sociocultural factors, the results showed that the longer the TAF, the greater the ASI and MTI prevalence. Hence, 12.9% and 3.2% of athletes who took, on average, <10 minutes to access their main training facility sustained an ASI or MTI, respectively. The ASI and MTI prevalence increased to 34.8% and 8.7%, 38.2% and 11.8%, and 52.6% and 15.8% among athletes who took, on average, between 10 and 19 minutes, between 20 and 39 minutes, and ≥40 minutes, respectively. Comparisons of the 95% CI showed that the prevalence of ASIs was statistically significantly lower for athletes who, on average, took <10 minutes to access their training facility compared to athletes in all other TAF categories.

The results also showed that athletes who took, on average, 15 minutes to prepare before and after training had the lowest ASI and MTI prevalence (13.3% and 3.3%, respectively), while those who took more time had a higher injury prevalence. Comparisons of the 95% CI showed that the prevalence of ASIs was statistically significantly lower for athletes with the lowest TPT compared to those in the other TPT categories.

Table 3 shows the bivariate associations between level of performance, sport, and impairment type. A greater proportion of Talent athletes had a visual impairment (30.4%) compared with all other impairment types. By contrast, a lower proportion of E1, E2–, and Mastery athletes had a visual impairment compared with all other impairment types (9.7%, 0.0%, and 0.0%, respectively). Table 4 shows the distribution of TAF and TPT by level of performance, sport, and impairment type.

**Table 3:** Level of Performance and Sport by Impairment Type^*a*^

	Limb Deficiency (n = 32)	Visual Impairment (n = 16)	Spinal Cord–Related Disorder (n = 43)	Central Neurological Disorder (n = 16)	Peripheral Neurological and Muscular Disorder (n = 13)	Unknown/ Other (n = 6)	All (n = 126)
Level of performance							
T	5 (21.7)	7 (30.4)	5 (21.7)	3 (13.0)	1 (4.5)	1 (4.3)	22 (100.0)
E1	8 (25.8)	3 (9.7)	10 (32.3)	5 (16.1)	4 (12.9)	1 (3.2)	31 (100.0)
E2–	10 (30.3)	0 (0.0)	12 (36.4)	5 (15.2)	5 (15.2)	1 (3.0)	33 (100.0)
E2+	6 (23.1)	6 (23.1)	10 (38.5)	1 (3.8)	2 (7.7)	1 (3.8)	26 (100.0)
M	3 (21.4)	0 (0.0)	6 (42.9)	2 (14.3)	1 (7.1)	2 (14.3)	14 (100.0)
Sport							
Athletics	6 (21.4)	8 (28.6)	6 (21.4)	7 (25.0)	1 (3.6)	0 (0.0)	28 (100.0)
Boccia	0 (0.0)	0 (0.0)	0 (0.0)	1 (33.3)	2 (66.7)	0 (0.0)	3 (100.0)
Cycling	8 (42.1)	1 (5.3)	3 (15.8)	1 (5.3)	5 (26.3)	1 (5.3)	19 (100.0)
Fencing	3 (25.0)	0 (0.0)	5 (41.7)	2 (16.7)	0 (0.0)	2 (16.7)	12 (100.0)
Five-a-side football	0 (0.0)	6 (100.0)	0 (0.0)	0 (0.0)	0 (0.0)	0 (0.0)	6 (100.0)
Powerlifting	1 (50.0)	0 (0.0)	0 (0.0)	0 (0.0)	0 (0.0)	1 (50.0)	2 (100.0)
Swimming	5 (62.5)	1 (12.5)	1 (12.5)	0 (0.0)	0 (0.0)	1 (12.5)	8 (100.0)
Rugby	2 (11.1)	0 (0.0)	13 (72.2)	2 (11.1)	1 (5.6)	0 (0.0)	18 (100.0)
Table tennis	3 (23.1)	0 (0.0)	7 (53.8)	1 (7.7)	1 (7.7)	1 (7.7)	13 (100.0)
Shooting	1 (11.1)	0 (0.0)	4 (44.4)	2 (22.2)	2 (22.2)	0 (0.0)	9 (100.0)
Archery	3 (37.5)	0 (0.0)	4 (50.0)	0 (0.0)	1 (12.5)	0 (0.0)	8 (100.0)

a Data are presented as n (%). E1, Elite 1; E2–, Elite 2–; E2+, Elite 2+; M, Mastery; T, Talent.

**Table 4:** Level of Performance, Sport, and Impairment Type by TAF and TPT^*a*^

	TAF				TPT			
	<10 min (n = 31)	10-19 min (n = 23)	20-39 min (n = 34)	≥40 min (n = 38)	15 min (n = 30)	30 min (n = 43)	1 h (n = 34)	≥1 h 30 min (n = 19)
Level of performance								
T (n = 22)	6 (27.3)	4 (18.2)	5 (22.7)	7 (31.8)	4 (18.2)	6 (27.3)	8 (36.4)	4 (18.2)
E1 (n = 31)	7 (22.6)	5 (16.1)	10 (32.3)	9 (29.0)	5 (16.1)	11 (35.5)	8 (25.8)	7 (22.6)
E2– (n = 33)	9 (27.3)	6 (18.2)	8 (24.2)	10 (30.3)	9 (27.3)	12 (36.4)	9 (27.3)	3 (9.1)
E2+ (n = 26)	3 (11.5)	5 (19.2)	9 (34.6)	9 (34.6)	8 (30.8)	11 (42.3)	4 (15.4)	3 (11.5)
M (n = 14)	6 (42.9)	3 (21.4)	2 (14.3)	3 (21.4)	4 (28.6)	3 (21.4)	5 (35.7)	2 (14.3)
Sport								
Athletics (n = 28)	8 (28.6)	3 (10.7)	8 (28.6)	9 (32.1)	9 (32.1)	8 (28.6)	9 (32.1)	2 (7.1)
Boccia (n = 3)	0 (0.0)	1 (33.3)	0 (0.0)	2 (66.7)	1 (33.3)	1 (33.3)	1 (33.3)	0 (0.0)
Cycling (n = 19)	11 (57.9)	1 (5.3)	2 (10.5)	5 (26.3)	4 (21.1)	6 (31.6)	4 (21.1)	5 (26.3)
Fencing (n = 12)	0 (0.0)	3 (25.0)	4 (33.3)	5 (41.7)	3 (25.0)	5 (41.7)	2 (16.7)	2 (16.7)
Five-a-side football (n = 6)	0 (0.0)	1 (16.7)	2 (33.3)	3 (50.0)	1 (16.7)	2 (33.3)	2 (33.3)	1 (16.7)
Powerlifting (n = 2)	1 (50.0)	1 (50.0)	0 (0.0)	0 (0.0)	1 (50.0)	1 (50.0)	0 (0.0)	0 (0.0)
Swimming (n = 8)	3 (37.5)	4 (50.0)	1 (12.5)	0 (0.0)	7 (87.5)	0 (0.0)	1 (12.5)	0 (0.0)
Rugby (n = 18)	1 (5.6)	4 (22.2)	7 (38.9)	6 (33.3)	2 (11.1)	9 (50.0)	5 (27.8)	2 (11.1)
Table tennis (n = 13)	4 (30.8)	3 (23.1)	3 (23.1)	3 (23.1)	2 (15.4)	4 (30.8)	5 (38.5)	2 (15.4)
Shooting (n = 9)	2 (22.2)	2 (22.2)	2 (22.2)	3 (33.3)	0 (0.0)	3 (33.3)	3 (33.3)	3 (33.3)
Archery (n = 8)	1 (12.5)	0 (0.0)	5 (62.5)	2 (25.0)	0 (0.0)	4 (50.0)	2 (25.0)	2 (25.0)
Impairment type								
Limb deficiency (n = 32)	10 (31.3)	10 (31.3)	5 (15.6)	7 (21.9)	14 (43.8)	8 (25.0)	4 (12.5)	6 (18.8)
Visual impairment (n = 16)	1 (6.3)	1 (6.3)	6 (37.5)	8 (50.0)	5 (31.3)	6 (37.5)	4 (25.0)	1 (6.3)
Spinal cord–related disorder (n = 43)	8 (18.6)	7 (16.3)	13 (30.2)	15 (34.9)	3 (7.0)	18 (41.9)	14 (32.6)	8 (18.6)
Central neurological disorder (n = 16)	4 (25.0)	1 (6.3)	6 (37.5)	5 (31.3)	2 (12.5)	7 (43.8)	6 (37.5)	1 (6.3)
Peripheral neurological and muscular disorder (n = 13)	6 (46.2)	1 (7.7)	3 (23.1)	3 (23.1)	2 (15.4)	3 (23.1)	6 (46.2)	2 (15.4)
Unknown/other (n = 6)	2 (33.3)	3 (50.0)	1 (16.7)	0 (0.0)	4 (66.7)	1 (16.7)	0 (0.0)	1 (16.7)

a Data are presented as n (%). E1, Elite 1; E2–, Elite 2–; E2+, Elite 2+; M, Mastery; T, Talent; TAF, time to access main training facility; TPT, time to prepare before and after training.

Table 5 shows the results of univariate analysis and multivariable logistic regression models to examine the relationship between TAF and TPT with injuries while controlling for level of performance, sport, and impairment type. As mentioned in the Methods section, to respect the rule of 10 events per variable, athletes classified into the unknown/other category were reclassified in the peripheral neurological and muscular disorder category, given that their ASI and MTI prevalence were equal (0.0% and 0.0%, respectively).^22^

**Table 5:** Univariate and Multivariable Binary Logistic Regression Analyses Results for Injuries^*a*^

	Univariate Analysis		Full Model^*b*^		Forward and Backward Model^*c*^	
	OR (95% CI)	*P*	OR (95% CI)	*P*	OR (95% CI)	*P*
TAF						
<10 min	Reference					
10-19 min	3.6 (1.0-15.4)	.064	3.4 (0.6-22.9)	.184	3.4 (0.7-19.8)	.157
20-39 min	4.2 (1.3-16.6)	**.026** ^*d*^	2.4 (0.5-13.8)	.314	2.5 (0.6-13.0)	.242
≥40 min	7.5 (2.4-29.2)	**.001** ^*e*^	5.5 (1.2-30.5)	**.036** ^*d*^	5.5 (1.3-27.3)	**.027** ^*d*^
TPT						
15 min	Reference					
30 min	5.1 (1.7-19.7)	**.008** ^*e*^	4.2 (1.0-22.4)	.069	4.4 (1.0-22.7)	.056
1 h	4.0 (1.2-16.0)	**.030** ^*d*^	3.6 (0.7-21.7)	.139	3.9 (0.8-21.5)	.100
≥1 h 30 min	5.9 (1.5-25.9)	**.012** ^*d*^	6.3 (1.1-44.4)	**.049** ^*d*^	6.4 (1.2-41.7)	**.038** ^*d*^
Akaike information criterion [area under curve]	NA		150.6 [0.83]		148.3 [0.83]	

a Bold values indicate a statistically significant difference. NA, not applicable; OR, odds ratio; TAF, time to access main training facility; TPT, time to prepare before and after training.

b The full model was adjusted for level of performance, sport, and impairment type.

c The forward and backward model was adjusted for sport and impairment type.

d *P* < .05.

e *P* < .01.

Athletes who took ≥40 minutes to access the main training facility were 5.5 times (95% CI, 1.2-30.5) more likely to have sustained an injury in the past 12 months compared with those who took <10 minutes. Similarly, athletes who took ≥1 hour and 30 minutes to prepare before and after training were 6.3 times (95% CI, 1.1-44.4) more likely to have had an injury in the past 12 months compared with those who took 15 minutes.

**Figure 1.**
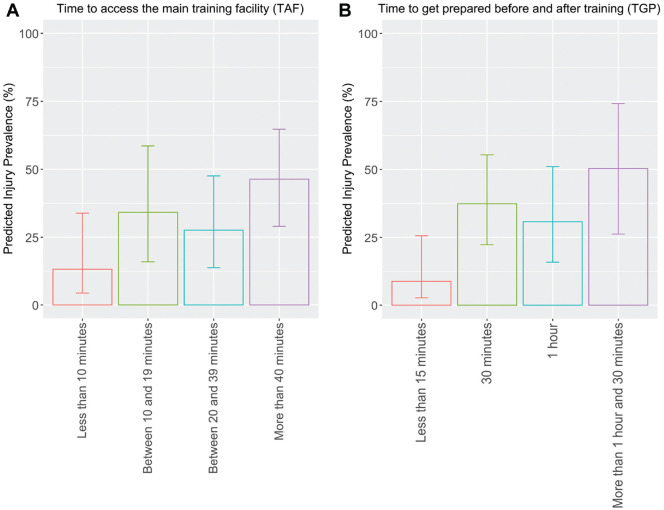
Predicted prevalence of injuries according to (A) time to access the main training facility and (B) time to prepare before and after training, averaged by sport and impairment type.

Figure 1 shows the predicted prevalence of injuries according to TAF from the multivariable logistic regression model using the forward and backward technique. It demonstrates that the predicted injury prevalence increases with TAF while controlling for other variables. The supplemental model assessing whether adding paraplegia and tetraplegia changed the results significantly showed no significant difference in the relationship between TAF, TPT, and the injury risk (Appendix Table A2).

## Discussion

Our main results from univariable and multivariable analyses (controlling for sport, impairment type, and level of performance) showed that athletes who reported a longer TAF and TPT were more likely to have sustained an injury in the past 12 months than those who reported a shorter TAF and TPT. For example, we found that the prevalence of injuries increased from <15% to >45% with either a longer TAF or TPT (Figure 1). To our knowledge, this is the first study that has investigated these factors in relation to injuries. Consistent with previous studies, the overall injury prevalence in our cohort was around 36%.^5,7,25^

We argue, in line with the HDM-DCP proposed by Fougeyrollas et al,^9,10^ that a longer TAF and TPT could serve as proxy measures for the relationship between Paralympic athletes’ personal factors (eg, level of impairment) and environmental factors (eg, accessibility). A longer TAF and TPT reflect the day-to-day challenges faced by Paralympic athletes related to transportation and preparation, which may be influenced by unfavorable social and environmental conditions. This, in turn, could lead to increased overall daily stress, potentially explaining the higher injury risk observed.^15,21,29^ The present study suggests that efforts to reduce travel and preparation constraints could lower the injury risk and provides initial empirical evidence of the HDM-DCP.

Additionally, descriptive analyses revealed that the group with the lowest injury risk (Mastery athletes and cycling athletes) also had the highest proportion of athletes with the shortest transportation time to their training facility. It is challenging to determine whether this lower injury risk is independently attributable to transportation time or related to intrinsic characteristics of the athletes or their sports.^16,24^ For Mastery athletes, this relationship might be caused by their individual attributes. These athletes have achieved significant success in international competitions, and their longevity at the highest level of performance might be associated with specific personal characteristics.^24^ Conversely, from a social perspective, in line with the HDM-DCP, it can be argued that their repeated sports achievements may have resulted in increased support from sports federations and sponsors. This support could have translated into better training conditions, such as shorter distances from home to the training facility and increased day-to-day assistance, which are reflected in the present study’s TAF and TPT. This opens avenues for future interdisciplinary research in sports medicine in which environmental, social, and individual factors could be considered together to propose a complex mechanism for sports injuries, in line with Wiese-Bjornstal’s^28^ biopsychosocial sports injury risk profile.

For example, future research may investigate the impact of TAF and TPT on physiological, biomechanical, or psychological variables that contribute to a higher risk of injuries. As noted, an increased TAF and TPT (related to personal and environmental factors) could lead to higher stress levels, potentially explaining the increased risk of injuries; however, this hypothesis requires further investigation.^15,29^

Additionally, we consider that the observed higher injury risk might be influenced by a third variable not controlled for in the present study: the level of impairment. Athletes with higher levels of impairment (in terms of functional capacity, which was not measured here) might fall into the higher TAF and TPT categories, raising questions about whether the level of impairment or TAF/TPT are independent explanatory factors for the injury risk. This remains to be evaluated, as it is also possible that athletes with higher levels of impairment receive more social support (eg, medical assistance, nursing) to help with everyday tasks. To our knowledge, no study has specifically investigated this factor, as most research has focused on impairment type without controlling for the level of impairment.^5,7^ This is an interesting perspective to explore in conjunction with TAF/TPT measures.

Other original, although more minor, findings were obtained from this study. In contrast to previous studies, five-a-side football players had a relatively low injury risk (16.7%). This injury risk was lower than those observed in athletics (57.1%), fencing (50.0%), or shooting (44.4%). These results are surprising because it is generally accepted that team/contact sports have a higher prevalence of injuries than individual/noncontact sports.^5,7,19^ These differences may have been caused by specific training conditions encountered by these athletes in their respective sports federations, which may differ from other countries.^28^ To test this hypothesis, a further cross-cultural study may compare training settings between countries and observe whether they are linked to a different injury prevalence. Further, concerning impairment types, the prevalence of ASIs was highest among athletes with a spinal cord–related disorder (48.8%), followed by those with a visual impairment (43.8%), a central neurological disorder (37.5%), or a limb deficiency (34.4%). The highest prevalence of ASIs observed among athletes with a spinal cord–related disorder and the lowest prevalence among those with a limb deficiency are in contradiction to the study by Fagher et al.^7^ However, when referring to MTIs, and not ASIs, a relatively similar pattern to that previous study was observed, but athletes with a visual impairment had the highest prevalence of MTIs (25.0%). These results showed that there is an interaction effect between impairment type and sport on the risk of injuries. Indeed, in five-a-side football, all athletes had a visual impairment, but this sport also had one of the lowest prevalence of injuries. Injured athletes with a visual impairment were thus probably involved in other sports including athletes with a visual impairment (athletics, cycling, and/or swimming). Further research could specifically investigate the interaction effects between sport and impairment type on the risk of injuries.

### Limitations

The main limitation of the present study is the use of a self-reported questionnaire to record athletes’ characteristics, injuries, and sociocultural factors. With regard to injuries, the latest consensus statement for the recording and reporting self-reported epidemiological data on injuries in sports was followed.^1,11^ The observed prevalence was consistent with previous studies conducted among Paralympic athletes.^7,25^ However, no detailed information regarding injuries was presented, which would be useful for improving the health of Paralympic athletes and the further development of injury prevention programs. Future epidemiological studies should report athletes’ injuries with the help of medical staff and using, for example, the web-based surveillance system developed by Derman et al.^6^

Regarding athletes’ characteristics, they were asked to report their sport, level of performance, and impairment type. To our knowledge, no previous study has conducted a sensitivity analysis to evaluate the potential discrepancy between the self-reporting of this information and the observed reality. In the absence of previous studies regarding these aspects, it is difficult to estimate the magnitude and direction of the bias. Meanwhile, in our opinion, the involvement of athletic staff members and sports federations as well as the high response rate obtained in this study provide partial evidence regarding the relative rigor of the answers reported by athletes. In this study, athletes were also invited to have someone of their choice help them in completing the questionnaire. This may have counterbalanced the relative difficulty that athletes with a specific medical condition might have had in reporting personal information. Nevertheless, further studies are needed to measure the bias in Paralympic athletes’ self-reporting of sport, level of performance, and impairment type.

With respect to the self-reporting of TAF and TPT, to our knowledge, this is the first time that these variables have been studied in relation to the risk of injuries. The use of this method could be considered a first step before the development of a more robust methodology to study these variables. Given the results observed, and their potential implications for athletes’ health and performance, future studies should be developed to more objectively assess these variables. These studies could, for example, use an accelerometer or a Global Positioning System device or involve a third person who could record a typical week of a Paralympic athlete.

An additional limitation of this study is the small sample size. Although the sample provides good generalizability within the French population of Paralympic athletes, given the high response rate (67.7%), it is not possible to determine whether these results are generalizable to the entire population of Paralympic athletes across different countries. Future research should involve longitudinal studies following Paralympic athletes over several years and across multiple countries and sports to enhance the generalizability of the findings.

## Conclusion

Our study demonstrated that Paralympic athletes who reported a longer TAF and TPT had a higher risk of injuries. Future studies are needed to understand the exact mechanism that would explain these results and to assist in the prevention of injuries in this population of athletes.

## Appendix

**Table A1:** Impairment Type by Categorization System

	Categorization System 1	Categorization System 2
Limb deficiency (n = 32)	Orthopaedic (n = 32), peripheral neurological and muscular disorder (n = 3), other neurological disorder (n = 2), intellectual and/or psychic (n = 1)	Left peroneal nerve palsy (n = 1), lower limb hemimelia (n = 8), upper limb hemimelia (n = 1), amputation (n = 11), limb length inequality (n = 8), autism spectrum disorder (n = 1)
Visual impairment (n = 16)	Visual impairment (n = 16)	Not applicable
Spinal cord–related disorder (n = 43)	Central neurological disorder (n = 34), peripheral neurological and muscular disorder (n = 7), other neurological disorder (n = 2), orthopaedic (n = 6)	Spinal cord injury leading to paraplegia (n = 15), spinal cord injury leading to tetraplegia (n = 21), left peroneal nerve palsy (n = 1), limb length inequality (n = 2)
Central neurological disorder (n = 16)	Central neurological disorder (n = 13), peripheral neurological and muscular disorder (n = 1), other neurological disorder (n = 1), orthopaedic (n = 1)	Stroke (n = 3), multiple sclerosis (n = 2), head injury (n = 2), cerebral palsy (n = 5)
Peripheral neurological and muscular disorder (n = 13)	Peripheral neurological and muscular disorder (n = 13), orthopaedic (n = 2)	Myopathy or spinal amyotrophy (n = 3), brachial plexus palsy (n = 3), left peroneal nerve palsy (n = 3), lower limb hemimelia (n = 1), amputation (n = 1), limb length inequality (n = 1)
Unknown/other (n = 6)	Peripheral neurological and muscular disorder (n = 1), other neurological disorder (n = 3), orthopaedic (n = 2)	Short stature (n = 1)

**Table A2:** Multivariable Binary Logistic Regression Analysis Results for Injuries With Paraplegia and Tetraplegia Added in Impairment Type^*a*^

	OR (95% CI)	*P*
Level of performance		
T	Reference	
E1	0.4 (0.1-2.0)	.278
E2–	0.5 (0.1-2.4)	.377
E2+	0.2 (0.0-1.1)	.073
M	0.1 (0.0-1.1)	.070
Sport		
Athletics/powerlifting	Reference	
Boccia/shooting/archery	0.1 (0.0-0.8)	**.039**
Cycling/swimming	0.0 (0.0-0.3)	**.002**
Fencing	0.3 (0.0-2.4)	.265
Five-a-side football/rugby	0.3 (0.1-1.4)	.134
Table tennis	0.1 (0.0-0.9)	**.040**
Impairment type		
Limb deficiency	Reference	
Visual impairment	0.3 (0.0-2.0)	.223
Paraplegia	2.6 (0.5-15.8)	.282
Tetraplegia	0.2 (0.0-1.4)	.117
Central neurological disorder	0.4 (0.1-1.9)	.240
Peripheral neurological and muscular disorder	0.0 (0.0-3.3E+20)	.990
Unknown/other	7.1 (0.6-201.7)	.158
TAF		
<10 min	Reference	
10-19 min	4.1 (0.6-31.0)	.146
20-39 min	1.9 (0.3-12.8)	.479
≥40 min	6.8 (1.4-42.7)	**.027**
TPT		
15 min	Reference	
30 min	4.6 (1.0-25.2)	.058
1 h	3.8 (0.7-23.5)	.133
≥1 h 30 min	6.1 (1.0-44.2)	.058
Akaike information criterion	146.6	

a Bold values indicate a significant difference. E1, Elite 1; E2–, Elite 2–; E2+, Elite 2+; M, Mastery; OR, odds ratio; T, Talent; TAF, time to access main training facility; TPT, time to prepare before and after training.

## References

[1] Bahr R Clarsen B Derman W , et al. International Olympic Committee consensus statement: methods for recording and reporting of epidemiological data on injury and illness in sport 2020 (including STROBE Extension for Sport Injury and Illness Surveillance (STROBE-SIIS)). Br J Sports Med. 2020;54(7):372-389. 32071062 10.1136/bjsports-2019-101969 PMC7146946

[2] Bahr R Krosshaug T. Understanding injury mechanisms: a key component of preventing injuries in sport. Br J Sports Med. 2005;39(6):324-329. 15911600 10.1136/bjsm.2005.018341 PMC1725226

[3] Bayram JM Hamilton DF Saunders DH. Epidemiology of American football injuries at universities in the United Kingdom. Orthop J Sports Med. 2020;8(10):2325967120960206. 10.1177/2325967120960206 PMC7607799 33195720

[4] Clarsen B Steffen K Berge HM , et al. Methods, challenges and benefits of a health monitoring programme for Norwegian Olympic and Paralympic athletes: the road from London 2012 to Tokyo 2020. Br J Sports Med. 2021;55(23):1342-1349. 34039584 10.1136/bjsports-2020-103717

[5] Derman W Runciman P Schwellnus M , et al. High precompetition injury rate dominates the injury profile at the Rio 2016 Summer Paralympic Games: a prospective cohort study of 51 198 athlete days. Br J Sports Med. 2018;52(1):24-31. 29030389 10.1136/bjsports-2017-098039

[6] Derman W Schwellnus M Jordaan E , et al. Illness and injury in athletes during the competition period at the London 2012 Paralympic Games: development and implementation of a web-based surveillance system (WEB-IISS) for team medical staff. Br J Sports Med. 2013;47(7):420-425. 23537560 10.1136/bjsports-2013-092375

[7] Fagher K Dahlström Ö Jacobsson J Timpka T Lexell J. Prevalence of sports-related injuries and illnesses in Paralympic athletes. PM R. 2020;12(3):271-280. 31260605 10.1002/pmrj.12211

[8] Fagher K Jacobsson J Timpka T Dahlström Ö Lexell J. The Sports-Related Injuries and Illnesses in Paralympic Sport Study (SRIIPSS): a study protocol for a prospective longitudinal study. BMC Sports Sci Med Rehabil. 2016;8(1):1-10. 27579170 10.1186/s13102-016-0053-x PMC5004301

[9] Fougeyrollas P. Documenting environmental factors for preventing the handicap creation process: Quebec contributions relating to ICIDH and social participation of people with functional differences. Disabil Rehabil. 1995;17(3-4):145-153. 7787197 10.3109/09638289509166709

[10] Fougeyrollas P Noreau L Boschen K. Interaction of environment with individual characteristics and social participation: theoretical perspectives and applications in persons with spinal cord injury. Top Spinal Cord Inj Rehabil. 2002;7(3):1-16.

[11] Gabbe BJ. How valid is a self reported 12 month sports injury history? Br J Sports Med. 2003;37(6):545-547. 14665599 10.1136/bjsm.37.6.545 PMC1724702

[12] Gulbin JP Croser MJ Morley EJ Weissensteiner JR. An integrated framework for the optimisation of sport and athlete development: a practitioner approach. J Sports Sci. 2013;31(12):1319-1331. 23631711 10.1080/02640414.2013.781661

[13] Hägglund M Waldén M Ekstrand J. Previous injury as a risk factor for injury in elite football: a prospective study over two consecutive seasons. Br J Sports Med. 2006;40(9):767-772. 16855067 10.1136/bjsm.2006.026609 PMC2564391

[14] Ivarsson A Johnson U Andersen MB Tranaeus U Stenling A Lindwall M. Psychosocial factors and sport injuries: meta-analyses for prediction and prevention. Sports Med. 2017;47(2):353-365. 27406221 10.1007/s40279-016-0578-x

[15] Johnson U Ivarsson A. Psychosocial factors and sport injuries: prediction, prevention and future research directions. Curr Opin Psychol. 2017;16:89-92. 28813363 10.1016/j.copsyc.2017.04.023

[16] Joncheray H Burlot F Besombes N Dalgalarrondo S Desenfant M. Performance factors and strategies favored by French Olympic athletes. Sociol Sport J. 2021;38(1):88-97.

[17] Lambert C Ritzmann R Akoto R , et al. Epidemiology of injuries in Olympic sports. Int J Sports Med. 2022;43(5):473-481. 34666411 10.1055/a-1641-0068

[18] Liampas A Neophytou P Sokratous M , et al. Musculoskeletal pain due to wheelchair use: a systematic review and meta-analysis. Pain Ther. 2021;10(2):973-984. 34387846 10.1007/s40122-021-00294-5 PMC8586284

[19] Luiggi M Griffet J. Sport injury prevalence and risk by level of play and sports played among a representative population of French adolescents: a school-based study. Rev Epidemiol Sante Publique. 2019;67(6):383-391. 31561942 10.1016/j.respe.2019.07.008

[20] Mwaka CR Best KL Cunningham C Gagnon M Routhier F. Barriers and facilitators of public transport use among people with disabilities: a scoping review. Front Rehabil Sci. 2024;4:1336514. 38283669 10.3389/fresc.2023.1336514 PMC10812606

[21] O’Connor DB Thayer JF Vedhara K. Stress and health: a review of psychobiological processes. Annu Rev Psychol. 2021;72:663-688. 32886587 10.1146/annurev-psych-062520-122331

[22] Peduzzi P Concato J Kemper E Holford TR Feinstein AR. A simulation study of the number of events per variable in logistic regression analysis. J Clin Epidemiol. 1996;49(12):1373-1379. 8970487 10.1016/s0895-4356(96)00236-3

[23] Rice SG. Medical conditions affecting sports participation. Pediatrics. 2008;121(4):841-848. 18381550 10.1542/peds.2008-0080

[24] Richard R Burlot F Duquesne V Joncheray H. “I had a dream: it was to play the games.” Sports socialisation processes of French Paralympic athletes. Eur J Sport Soc. 2022;19(2):99-116.

[25] Steffen K Clarsen B Gjelsvik H , et al. Illness and injury among Norwegian Para athletes over five consecutive Paralympic Summer and Winter Games cycles: prevailing high illness burden on the road from 2012 to 2020. Br J Sports Med. 2022;56(4):204-212. 34607800 10.1136/bjsports-2021-104489

[26] Van Mechelen W Hlobil H Kemper HCG . Incidence, severity, aetiology and prevention of sports injuries. Sports Med. 1992;14(2):82-99. 1509229 10.2165/00007256-199214020-00002

[27] Whittaker JL Small C Maffey L Emery CA. Risk factors for groin injury in sport: an updated systematic review. Br J Sports Med. 2015;49(12):803-809. 25833903 10.1136/bjsports-2014-094287

[28] Wiese-Bjornstal DM. Psychology and socioculture affect injury risk, response, and recovery in high-intensity athletes: a consensus statement. Scand J Med Sci Sports. 2010;20(Suppl 2):103-111. 10.1111/j.1600-0838.2010.01195.x 20840568

[29] Williams JM Andersen MB. Psychosocial antecedents of sport injury: review and critique of the stress and injury model. J Appl Sport Psychol. 1998;10(1):5-25.

